# Supplementary figures and images for: How mangabey molar form differs under routine vs. fallback hard-object feeding regimes
Source: PeerJ. 2023 Dec 11;11:e16534. doi: 10.7717/peerj.16534 (PMC10720418; doi:10.7717/peerj.16534)

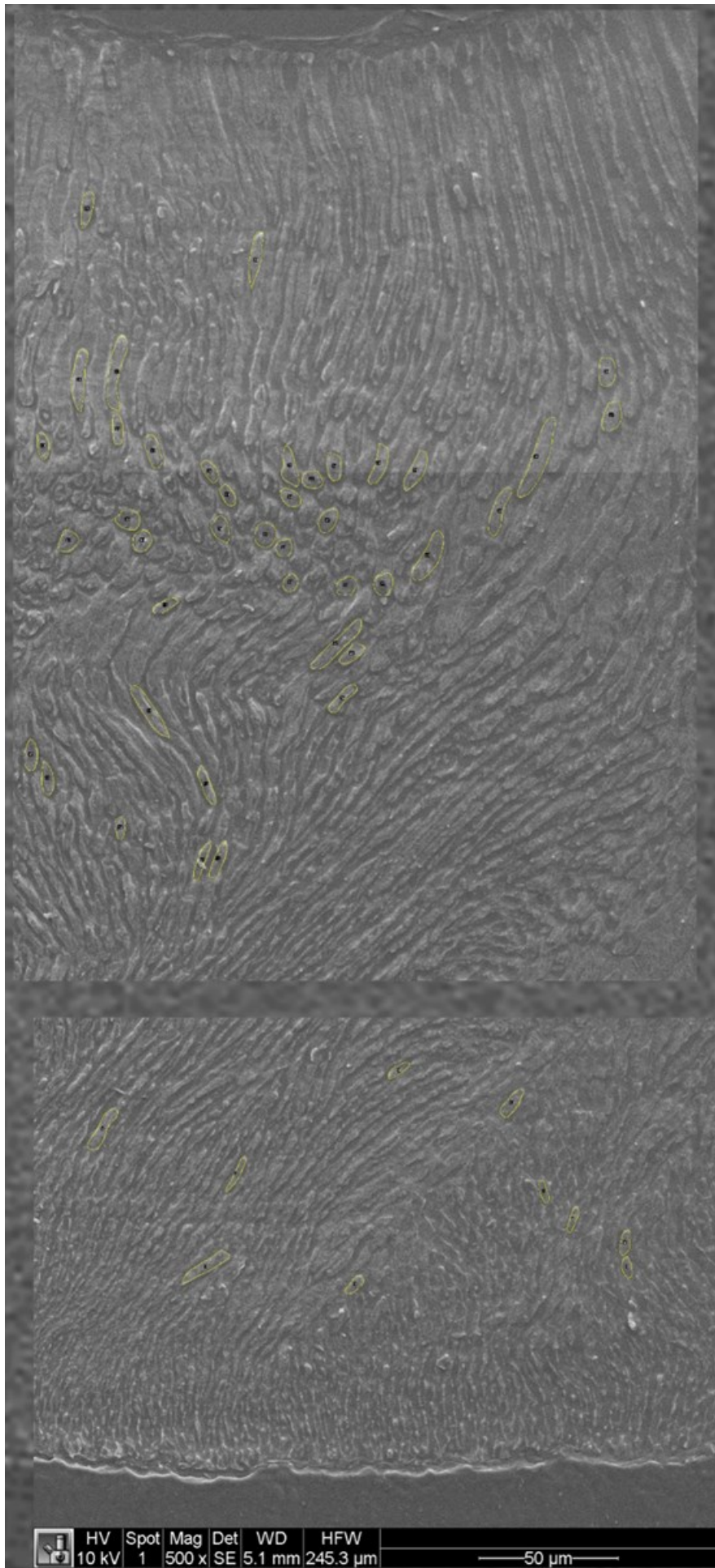

Supplement: Figure S1 [file peerj-11-16534-s001.pdf]

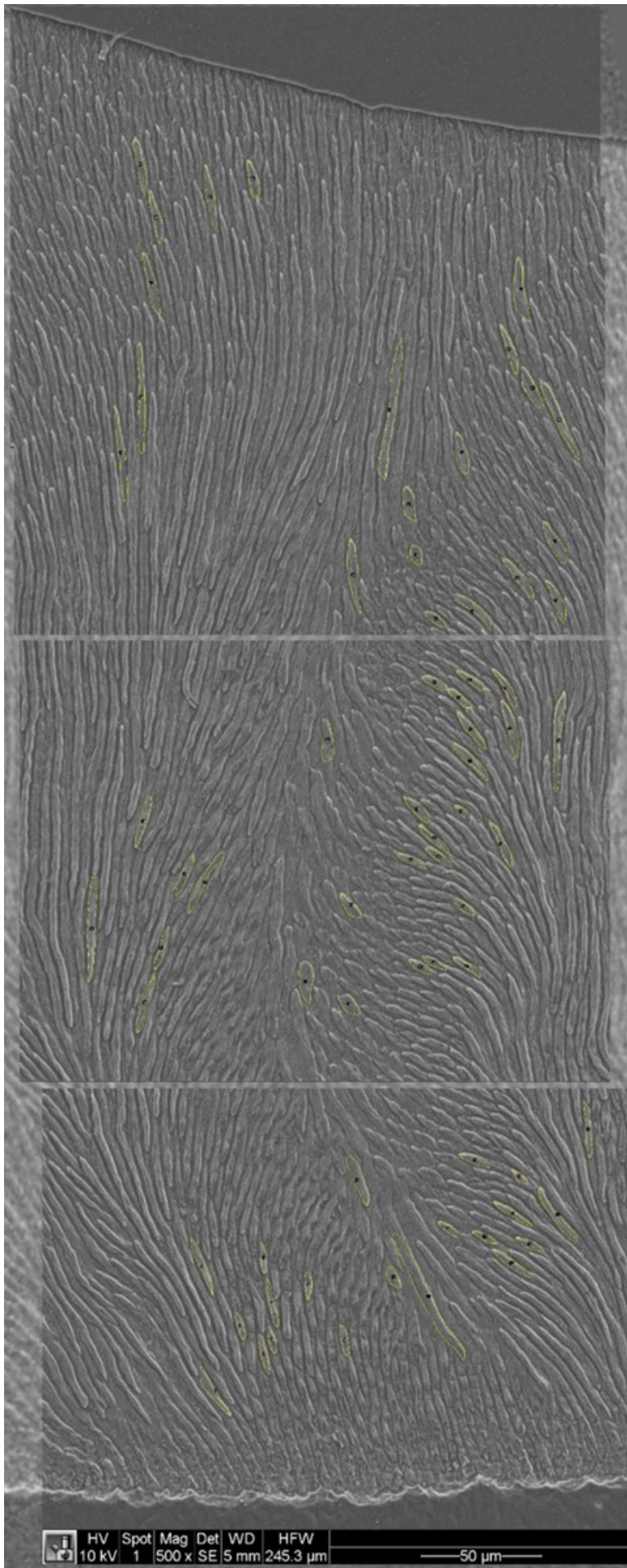

Supplement: Figure S2 [file peerj-11-16534-s002.pdf]

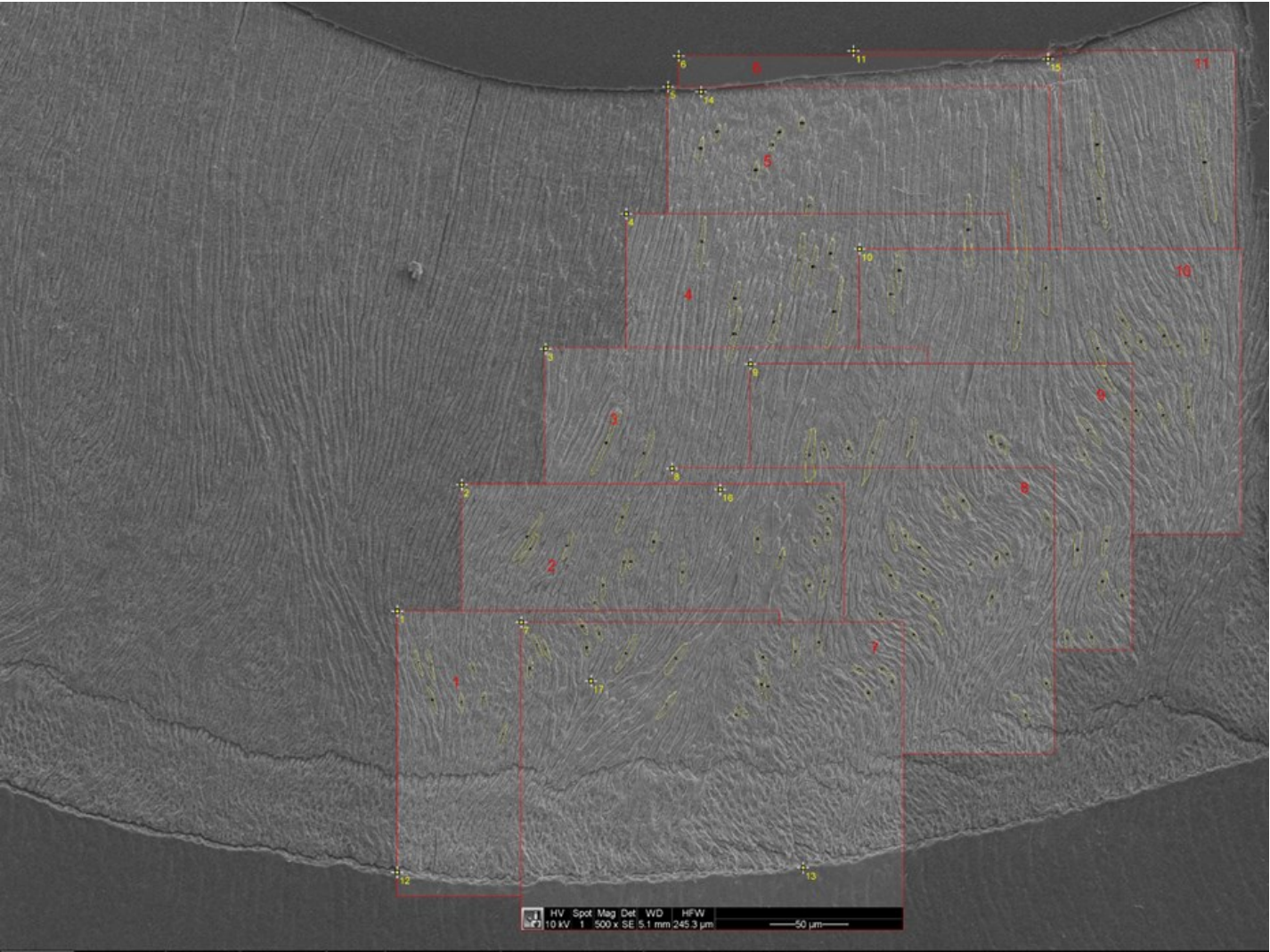

Supplement: Figure S3 [file peerj-11-16534-s003.pdf]

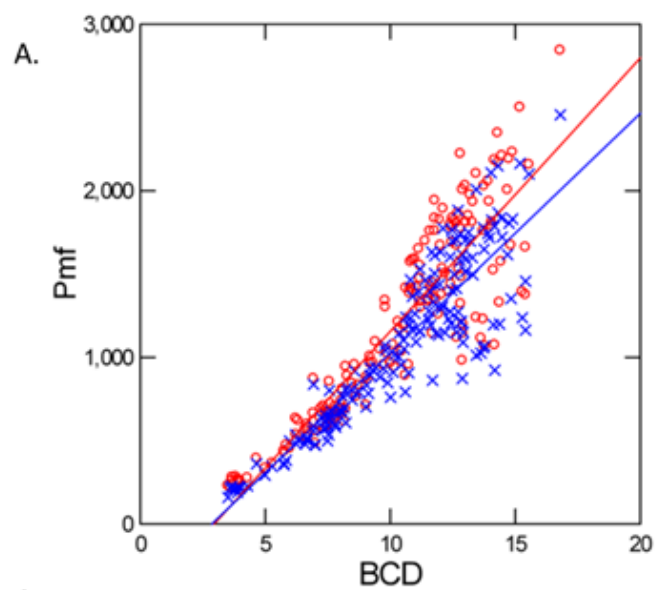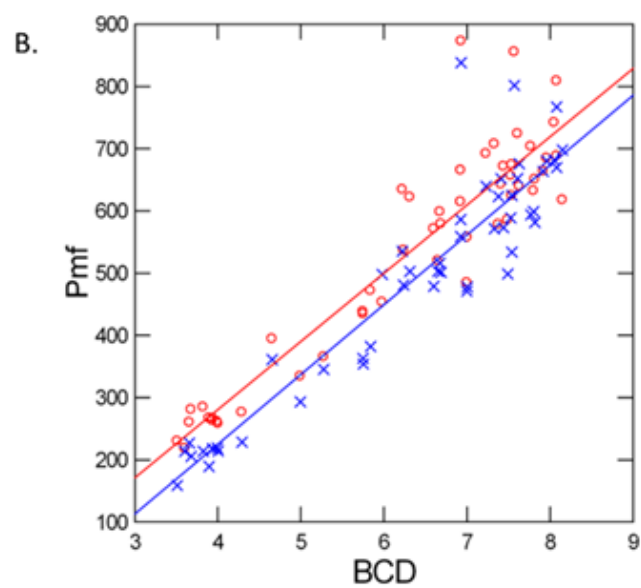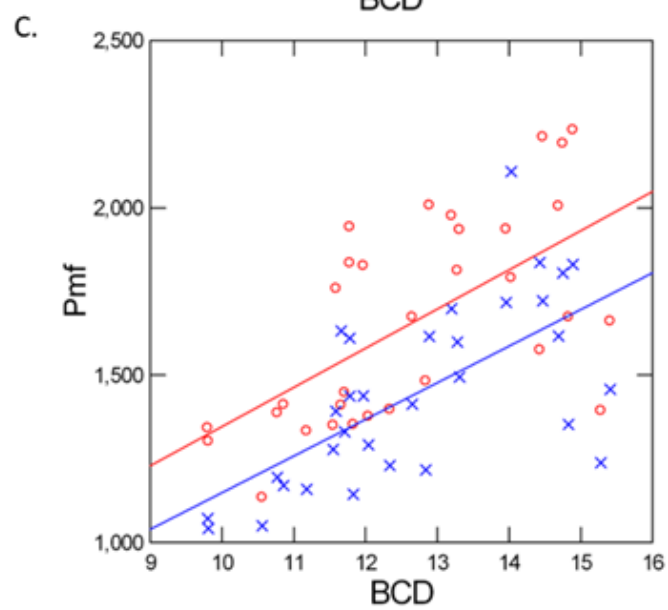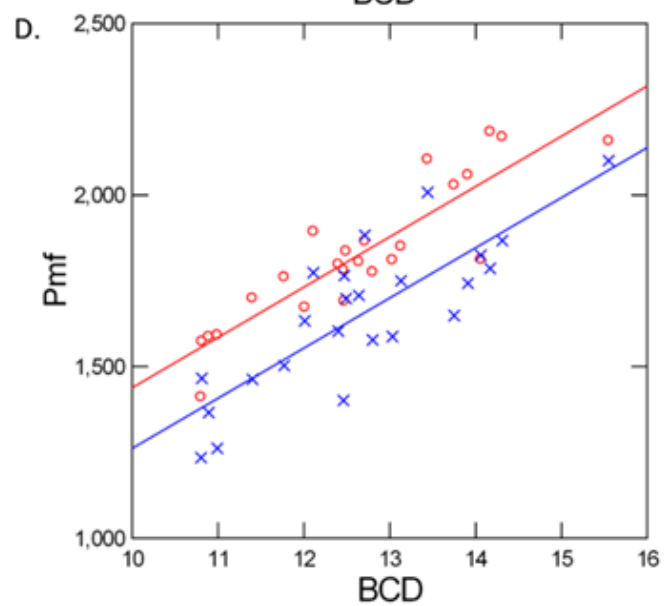

○ PMF\_PROTOCONID  
× PMF\_METACONID

Supplement: Figure S4 — A. Across living and extinct taxa (N=170), PMF of the protoconid increases more steeply with molar size than does PMF of the metaconid; the reason for this apparent allometric relationship is not currently clear, but it might reflect dietary shifts across species as body size increases. Data plotted in A are from the current study combined with data from Schwartz et al., 2020 SI (sample composition: Cercopithecus (N=11), Lophocebus albigena (N=9), Cercocebus atys (N=11), Pongo (N=9), Pan troglodytes (N=16), Pan paniscus (N=8), Homo sapiens (N=9), Gorilla (N=13), Australopithecus anamensis (N=13), Australopithecus africanus (N=32), Australopithecus afarensis (N=8), Australopithecus robustus (N=24), and Australopithecus boisei (N=7) B. A subset (N=40) of the data from the larger sample (N=170) selected for BCDs less than 8.2 mm, the largest BCD of any molar in the current study. Note that in this molar size range, the rate of increase for the protoconid is similar to that of the rate of increase for the metaconid (subset sample composition: Cercopithecus (N=11), Lophocebus albigena ( N=9), Cercocebus atys (N=11), Pan troglodytes (N=5), Pan paniscus (N=2), Homo sapiens (N=2). C and D. Two species with sample sizes greater than 20 —A. africanus (C) and A. robustus (D) —were selected to explore rates of change of PMF of the protoconid and metaconid within a species. A. africanus and A. robustus both appear to have similar rates of change in PMF with BCD. Altogether, these graphs suggest that across species, PMF of the protoconid increases at a faster rate that PMF of the metaconid, most notably in species with larger molars, but that within species, PMF of the protoconid and metaconid scale in a similar way with tooth size. [file peerj-11-16534-s004.pdf]
